# Cowden disease: what is in a name?

**DOI:** 10.1007/s12055-025-02151-y

**Published:** 2025-12-26

**Authors:** Randolph Hung-Leung Wong

**Affiliations:** Division of Cardiothoracic Surgery, Department of Surgery, Prince of Wales Hospital, The Chinese University of Hong Kong, Sha Tin, Hong Kong

**Keywords:** Eponymn, Genetic disease, Aortic aneurysm

“So long as signs are determined from fancied analogies, and named from these or after the person who describes them, there cannot but be obscurity and confusion.”

—Austin Flint (1812–1886)

Austin Flint’s prescient warning, articulated over a century ago, resonates with particular urgency in our genomic era. In this issue of the Journal, Albrecht et al. presented a rare case of ascending aortic aneurysm in a 4-year-old boy with a maternal history of Cowden syndrome, and an aortic root replacement was performed with a good outcome [1]. The patient was found to have a Phosphatase and TENsin homolog (PTEN) gene mutation. Given the atypical vascular presentations and in the absence of other features such as hamartoma, it is difficult to conclude whether this patient really suffered from Cowden syndrome. This case exemplifies two pivotal challenges in contemporary medicine: elucidating the molecular basis of PTEN-related disease and abandoning the antiquated practice of eponymous nomenclature.

## The PTEN paradigm and descriptive precision

Cowden syndrome—an eponym itself—results from PTEN tumor suppressor gene mutations that disrupt fundamental cellular regulation. The PTEN protein operates primarily as a lipid phosphatase, antagonizing the Phosphoinositide 3-kinase (PI3K)/Protein Kinase B(AKT)/Mammalian Target of Rapamycin (mTOR) pathway by converting phosphatidylinositol-3,4,5-trisphosphate to phosphatidylinositol 4,5-bisphosphate. This enzymatic action functions as a critical “brake” on cell proliferation, promoting apoptosis in damaged cells. Beyond this canonical role, PTEN’s weaker protein phosphatase activity modulates cell migration through substrates like focal adhesion kinase, while its phosphatase-independent nuclear functions maintain genomic stability via centromere protein C binding and deoxyribonucleic acid (DNA) repair coordination. These pleiotropic mechanisms explain the multisystem manifestations—including increasingly recognized vascular pathologies such as aortic aneurysm—that characterize PTEN mutation spectrum disorders.

This molecular complexity renders the eponym “Cowden syndrome” not merely uninformative but actively detrimental to clinical reasoning. As Flint observed, naming diseases after individuals inevitably injects obscurity into medical communication. A genetics-based designation like “PTEN hamartoma tumor syndrome” immediately conveys etiology, pathophysiology, and therapeutic implications—a scientific signpost rather than a historical footnote. The World Health Organization’s (WHO) 2015 nomenclature guidelines explicitly prohibit personal, geographic, or species names to prevent stigma and facilitate global standardization. Yet practical barriers persist: inconsistent possessive usage, regional nomenclature variations, and the frequent association of single individuals with multiple disparate conditions create confusion that undermines literature searches, international collaboration, and clinical trial enrollment.

## Establishing genetic causality: rigorous standards

Confirming PTEN’s role in aortopathy requires systematic evidence collection under American College of Medical Genetics and Genomics and the Association for Molecular Pathology (ACMG/AMP) frameworks. Clinical validation demands multi-generational segregation analysis demonstrating variant co-inheritance with disease; significantly elevated prevalence in cases versus population controls; de novo occurrence in dominantly inherited conditions; and phenotype specificity such as early-onset dissection (< 50 years) or associated syndromic features. Functional substantiation necessitates in vitro or in vivo assays confirming deleterious effects on protein function, stability, or localization in vascular smooth muscle cells; animal models exhibiting aortopathy phenotypes; and computational predictions consistently supporting pathogenicity. Only through such rigorous, multi-domain evidence integration can expert panels like the Clinical Genome Resource (ClinGen) Variant Curation Expert Panel (VCEP) classify variants as Pathogenic, Likely Pathogenic, or Variant of Uncertain Significance—classifications that directly inform patient management and cascade screening.

## Conclusion

Flint’s nineteenth-century insight anticipated our twenty-first-century imperative: disease nomenclature must facilitate, not obstruct, scientific clarity. While the authors correctly highlight vascular surveillance in PTEN mutation carriers, the broader mandate is clear—we must complete the transition from eponym-laden observational taxonomy to mechanism-based descriptive terminology. In an era where genomic diagnostics and precision therapeutics define standard of care, applying Flint’s principle means ensuring disease names illuminate molecular pathways rather than memorialize historical figures. The scientific signposts we establish today will guide the clinical decisions of tomorrow.
